# What Do We Know about the Long-Term Course of Early Onset Bipolar Disorder? A Review of the Current Evidence

**DOI:** 10.3390/brainsci11030341

**Published:** 2021-03-08

**Authors:** Carlotta Cirone, Ilaria Secci, Irene Favole, Federica Ricci, Federico Amianto, Chiara Davico, Benedetto Vitiello

**Affiliations:** 1 Child and Adolescent Neuropsychiatry—Department of Public Health and Pediatric Sciences, Universita’ degli Studi di Torino, 10126 Turin, Italy; carlotta.cirone@unito.it (C.C.); ilaria.secci@unito.it (I.S.); irene.favole@unito.it (I.F.); federica.ricci@unito.it (F.R.); benedetto.vitiello@unito.it (B.V.); 2 Child and Adolescent Neuropsychiatry—Department of Neurosciences, Universita’ degli Studi di Torino, 10126 Turin, Italy; federico.amianto@unito.it; 3 Department of Mental Health, Johns Hopkins Bloomberg School of Public Health, Baltimore, MD 21205, USA

**Keywords:** bipolar disorder, children, adolescents, long-term outcome

## Abstract

Aim: Early onset of psychopathology is often an index of a more severe clinical course and worse prognosis. This review examined the course of bipolar disorder (BD) with onset in childhood and adolescence, with a focus on persistence of symptoms, severity of illness, comorbidity, and functional impairment. Methods: The databases of PubMed, Embase, and PsycInfo were systematically searched for publications since 1990 reporting on long-term (12 months or longer) assessments of patients with early onset BD. Results: Forty-two relevant publications were identified, which reported on data derived from 15 different patient cohorts, including 7 prospective research psychopathology studies, 4 medical record reviews, 2 follow-ups of clinical trial samples, 1 managed care database, and 1 nationwide registry, for a total of 10,187 patients. The length of follow-ups ranged from 1.0 to 15 years. Diagnostic stability of BD ranged from 73% to 100% over ten years. Recovery rate from an index episode was 81.5–100% and recurrence rate was 35–67%. Suicide attempt cumulative prevalence in five years was 18–20%. Earlier age at the first episode predicted a more severe clinical course. Conclusions: Early onset BD persists over time through adolescence, with homotypic diagnostic continuity over the years, but heterogeneity in the severity of the clinical course. Whether early identification and treatment improves distal prognosis remains to be further investigated.

## 1. Introduction

Bipolar disorder (BD) is a chronic condition and a leading cause of morbidity and mortality, being associated with reduced life expectancy primarily due to cardiovascular disease and suicide [1]. Even though effective treatments exist and extended remission is possible, BD is a lifelong condition for which a definitive cure is not yet available. Lifetime prevalence is estimated to be about 1% worldwide [2], but rates up to 2.6% have been reported [3,4]. Based on retrospective reports, the mean age of onset of BD type I (BD-I) is around 18 years [5], with a peak in the incidence of the first manic episode between 21 and 23 years [3,6]. One study identified three peaks of onset in life, at 17, 26, and 35 years, respectively [7]. Thus, when also considering that a depressive episode can precede mania as the initial manifestation, it should be noted that onset of BD in adolescence is not uncommon.

Estimates of the prevalence of early onset BD (i.e., with onset under the age of 18 years) are variable. One epidemiological study in adolescents found a prevalence of 1.1% [8]. A subsequent meta-analysis of 12 epidemiological studies yielded a lifetime prevalence up to age 21 of 1.8% (95% CI, 1.1–3.0%) [9], but rates as high as 2.5% at age 18 have been reported [10]. The apparent incongruence between adult and adolescent prevalence rates is likely due to methodological differences in ascertainment. For years, controversy surrounded the diagnosis of BD in children with regard to whether extreme chronic irritability represented a BD manifestation [11]. There is now general acceptance that one should apply to children and adolescents the same diagnostic criteria as in adults, requiring the presence of discrete episodes of persistently abnormal mood elevation for a diagnosis of BD [5,12].

Long-term follow-up studies are especially useful for understanding developmental psychiatric phenomenology and linking child to adult psychopathology. By delineating the trajectory of the disorder and documenting its evolution in time, prospective studies can clarify if and to which extent a diagnosis of early onset BD (i.e., under the age of 18 years) is predictive of adult BD. These studies also help document the impact of the disorder on highly relevant outcomes, such as educational attainment, social functioning, medical complications, suicidal behavior, and hospitalization [13,14,15,16,17]. A growing body of research suggests that early onset BD is associated with a more severe course of illness, increased risk of suicidality and comorbid psychopathology, and worse functional outcomes (e.g., academic achievement, employment, living independently, marriage, and children) [9,18,19,20]. Some recent reviews reported that the most prevalent comorbid diagnoses in early onset BD are attention-deficit hyperactivity disorder (ADHD), oppositional defiant disorder/conduct disorder, substance use disorders, and anxiety disorders [11,12]. ADHD is more common in childhood, while comorbidity in late adolescence is similar to that in adulthood, with higher rates of substance abuse and anxiety disorders [12,21]. Comorbid ADHD or anxiety have been found to be associated with more severe mood symptoms, greater functional impairment, and worse clinical course [9,18].

We conducted a systematic review of prospective follow-up studies of early onset BD with the aim of evaluating the currently available evidence for diagnostic continuity, symptom persistence, and impact on functional outcomes. We did not include studies reporting on retrospectively derived child psychopathology obtained from patients first diagnosed with BD as adults because of the risk of selection and recall biases. To our knowledge, no similar systematic review had been published. The main questions guiding this systematic review were: (1) what is the stability of BD with onset in children and adolescents? Is there a homotypic or heterotypic continuity? (2) What are the outcomes for children and adolescents with BD in terms of functional outcomes, suicidality, hospitalization, response to treatment, and comorbidity? (3) Does early onset BD have a more severe clinical course than later onset BD? (4) Does early treatment lead to a more favorable outcome in adulthood?

## 2. Materials and Methods

### 2.1. Literature Search

A systematic review of relevant publications was conducted in accordance with the Preferred Reporting Items for Systematic Reviews and Meta-Analyses (PRISMA) statement [18]. A literature search was conducted electronically in three databases (PubMed, Embase, and PsycInfo) on 13 November 2020. The search terms were: “bipolar disorder” or “mania” and “child” or “adolescent” and “outcome” or “prognosis” or “course” or “longitudinal”, with a time filter for date of publication set to after 1989. The time frame was limited to the last 30 years because the diagnostic construct of bipolar disorder was previously infrequently applied to children, but has become increasingly used since the 1990s [11]. The complete search string is available in Supplementary Table S1.

### 2.2. Selection of Relevant Publications

After eliminating duplicate records, publication titles and abstracts were screened according to the following eligibility criteria:

Inclusion criteria: (a) peer-reviewed publications; (b) longitudinal studies of children and adolescents (up to 18 years of age) with a diagnosis of BD based on an internationally accepted definition; (c) for studies in which only some of the participants had a diagnosis of BD, the data on BD cases had to be reported separately; (d) minimum sample size of 30 patients with a diagnosis of BD; and (e) a minimum follow-up time of one year.

Exclusion criteria: (a) studies published before 1990; (b) publications in languages other than English; (c) reviews, meta-analyses, clinical trial reports, clinical case reports, editorials, commentaries, book chapters, and conference presentations.

### 2.3. Data Collection and Analysis

First, four reviewers (CD, IS, CC, and IF) independently evaluated the titles and abstracts of the first 300 and last 300 publications (by date of publication) that were identified with the electronic searches, with a rate of agreement of 86.2% [22] and consensus agreement on the remaining after group discussion. Then, the reviewers independently screened the remaining publications and selected the possibly relevant studies; in cases of doubt, each paper was discussed collectively among the reviewers. The full texts of the selected articles were then screened independently by two teams of two reviewers each (CC/IF and CD/IS). All papers with doubtful assignments were re-evaluated collectively to reach consensus agreement, with external supervision (BV). The data received a descriptive and qualitative analysis focused on the main questions that drove the review.

### 2.4. Risk-of-Bias Appraisal

The methodological quality of the included studies was assessed by three independent reviewers who reached agreement using a risk-of-bias assessment checklist (Supplementary Table S2) based on the Newcastle Ottawa Scale (NOS) for assessing the quality of non-randomized studies in meta-analyses [23]. The checklist included individual items about the study external (sample representativeness and use of validated diagnostic interviews) and internal validity (blind raters, adequate sample size, and sample retention). The risk of bias for each item was scored as low, moderate, or high. Each study was then given an overall score corresponding to the worst score received on among the item scores.

## 3. Results

### 3.1. Selected Publications

**Figure 1:**
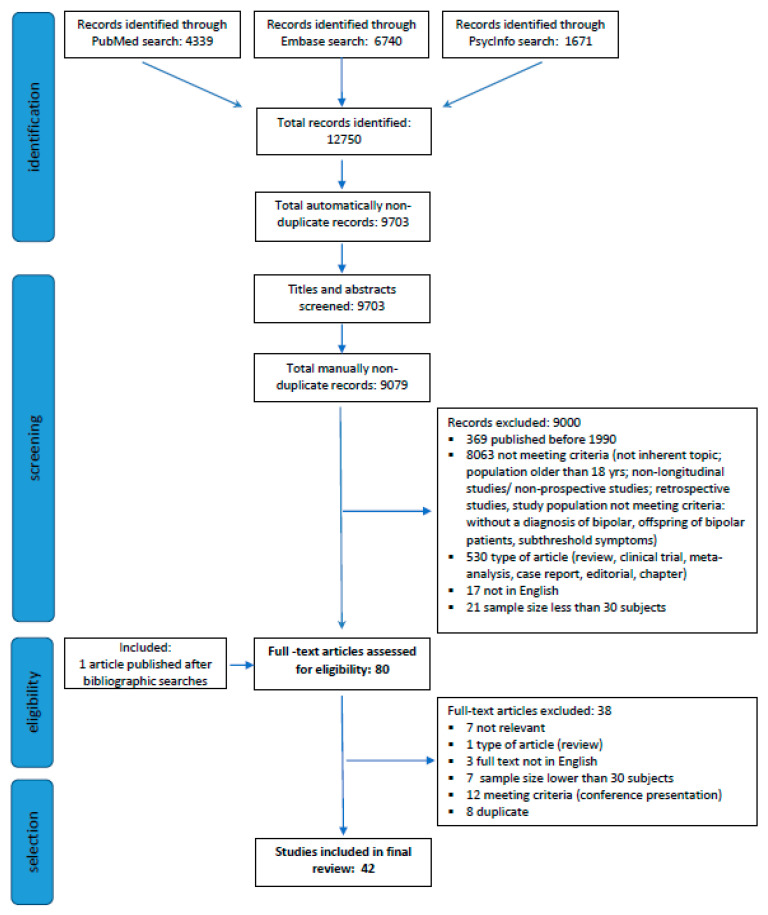
Review flowchart.

The search yielded a total of 12,750 records (Figure 1). After electronically eliminating duplicates, the titles and abstracts of the remaining 9703 records were manually screened for suitability based on the stated inclusion and exclusion criteria. Based on this review, another 624 duplicate publications were identified and therefore discarded. One article published after the bibliographic searches was added [17]. Among the remaining records, 79 were identified for possible inclusion, and their full texts were further evaluated for relevance. A total of 42 publications, reporting on 15 different patient cohorts, were eventually included in the systematic review (Table 1). The characteristics of each publication are summarized in Supplementary Table S3.

According to the risk-of-bias analysis, of the 42 selected studies, three were deemed to be at low risk of bias for all the criteria and were determined to be of high quality. Twenty-six studies demonstrated moderate risk of bias in at least one of the criteria. Thirteen studies were judged to be at high risk of bias in at least one of the criteria. The most common risk of bias was lack of a blind rater, followed by lack of validated diagnostic interviews. Sample retention was very good (>80%) in most of the studies (Supplemental Table S3).

There were important differences in design and methods among the 15 identified patient cohorts. Some were samples of convenience, whose medical records had been prepared for clinical or administrative purposes and then analyzed by researchers to reconstruct the course of illness. Others were ad hoc research samples, specifically recruited for assessing psychopathology over time. Seven were prospective research assessments of patients using validated diagnostic and assessment research instruments (Table 1) [14,15,16,24,25,27,57]. Cumulatively, these cohorts included 956 patients with periods of observation between 1.0 and 12.5 years. Four cohorts were based on reviews of medical records from naturalistically treated patients, with a total of 363 patients and a period of observation between 1.5 and 15 years [29,30,53,55]. Two cohorts consisted of follow-up of clinical trial samples, for a total of 220 patients and a period of observation of two years [56,60]. One was a large managed care insurance database, which included 8129 patients and a period of observation of 1.4 years [52], and one was a nationwide population registry in Denmark, which included up to 519 patients over a 10-year period (Table 1) [59]. The large majority of the cohorts were assessed using the DSM criteria, especially DSM-IV. Only two used ICD-10 criteria [52,59] and one used the Research Diagnostic Criteria [24]. Most of the study cohorts were from the USA (11 out of 15), two were from Europe, including a nationwide registry database, and two were from India.

### 3.2. Diagnostic Stability

Based on medical record reviews, 95.8% of the patients retained a BD diagnosis, with all BD-I patients maintaining their diagnosis and about half of BD-NOS patients converting to BD-I [54,55]. Among patients with multiple hospitalizations, 73% had diagnostic concordance between the first and second hospitalization [30]. Among the research prospective studies, the *Course and Outcome of Bipolar Youth* (COBY) found substantial diagnostic continuity of BD. There were diagnostic changes but within the BD spectrum, with 25% of BD-II converting to BD-I and 38% of BD-NOS converting to BD-I or BD-II over four years [14]. Exposure to antimanic medication was associated with a lower risk of conversion to BD-I [14]. Likewise, follow-up studies of clinical trial samples reported that 24% of patients with BD-NOS converted to BD-I [57].

In the *Phenomenology and Course of Pediatric Bipolar Disorders* study, the BD-I diagnosis had 100% continuity over an eight-year follow-up [27]. Another research prospective study reported that 73.1% of the patients continued to meet diagnostic criteria for BD-I [16].

Analysis of the Danish population database found that the stability of the diagnosis was 86% at three years and 73% at ten years [59]. By ten years, 17% had changed diagnosis to schizophrenia. The diagnostic change was more likely in patients who were males, had been diagnosed during a hospitalization, had abused substances, were given a previous diagnosis of schizophrenia spectrum disorder, or had parents with schizophrenia spectrum disorder [59].

### 3.3. Course of Illness

Rates of recovery (generally defined as euthymia for at least eight consecutive weeks) from index episode between 81.5% and 100% were reported [14,15,24,25,27,29,52]. Time to recovery was reported to be 36.0 weeks (SD 25.0) in one study [26], and 20 weeks (SD 13) in another [15]. Recovery time was found to be shorter when the index episode was manic (median 8 weeks) or mixed (11 weeks) rather than depressed (26 weeks) [24]. Predictors of recovery were more prolonged lithium treatment and living in an intact biological family [26,28]. In a one-year follow-up of hospitalized adolescents with first episode BD mania, functional recovery was 39%, as compared with the 85% symptomatic recovery rate [15].

A relapse (recurrence) rate of 62.5% at 1.5 years was reported in the COBY [14], and of 73% during the eight years of the *Phenomenology and Course of Pediatric Bipolar Disorders* study [27]. Other studies reported relapse rates from 35% to 67% at five years [24,25,29].

The course of illness was characterized by presence of mood symptoms for most of the time [27,31]. In one study, only 6.4% were euthymic after a four-year follow-up [16]. In a medical record review on a Spanish sample, children with BD had a chronic course with little inter-episodic recovery [54]. However, in a prospective follow-up of a clinical trial sample, a substantial proportion (25–30%) of youth with bipolar I or II disorder maintained euthymic states over extended periods [60].

In COBY, four different longitudinal mood trajectories were identified with latent class analysis: “predominantly euthymic” (24.0%), “moderately euthymic” (34.6%), “ill with improving course” (19.1%), and “predominantly ill” (22.3%) [38].

Early age at onset was found to predict a more severe course of illness [13,27,31,38,54]. Other negative prognostic predictors were family history of BD or substance abuse, sexual abuse, suicidality, more severe mood symptoms, and presence of psychotic symptoms [14,38,46]. Rates of psychosis of 40% and 61% were reported [28,53]. Psychosis was associated with a more severe symptomatic course and higher rates of suicide attempt and hospitalization [46].

Social and school impairment often persisted after symptomatic recovery. In COBY, 40.5% of the patients were impaired in interpersonal functioning and 92.8% in school activities [49]. A decline in psychosocial functioning was found to precede onset of depressive episodes [49]. No decline in cognitive functioning over time was detected within the 2.5 years of follow up [42]. Lithium therapy was associated with better psychosocial functioning over a ten-year period [48]. At least three studies reported an association of better outcomes with better quality of family relationships [27,51,56].

### 3.4. Suicidal Behavior

In COBY, 18% of the patients made a suicide attempt during a five-year period of observation [33]. Females were more likely to attempt suicide. The suicidality risk was greater among patients with more severe depression and family history of depression [33]. A 20% rate for suicide attempts was reported in another five-year prospective research follow-up study [24]. In a medical record review, 57% of the patients were found to have suicidal ideation and/or made attempts at suicide [53].

### 3.5. Comorbidity

A high rate of comorbidity with attention deficit/hyperactivity disorder was documented. In COBY, 58.6% of the sample met criteria for ADHD at study entry [31]. An anxiety disorder was present in most patients, persisted in time, and was associated with more recurrences of mood episodes and less time spent in euthymia [37,39].

The course of BD was associated with a substance abuse disorder in about a third of the cases: 35% of the patients developed a substance abuse disorder by early adulthood in the *Phenomenology and Course of Pediatric Bipolar Disorders* study [27] and 32% in COBY [34]. In COBY, 12.2% of the patients met criteria for borderline personality disorder [40].

## 4. Discussion

This systematic review of studies reporting on the longitudinal course of BD in children and adolescents identified a substantial number of informative reports. The studies used different approaches, including recruitment of well-characterized research samples specifically aimed at assessing developmental psychopathology, convenience samples from clinical trials that received extensive follow-up, and reviews of naturalistically collected medical and administrative records. Considering the diversity of the studies and the heterogeneity of methods, there is a remarkable consistency in the results and convergence on several important issues.

There is evidence of diagnostic stability of BD over a period up to about ten years [55,59]. Stability was especially good within the BD spectrum, with a tendency for BD-NOS to convert to BD-I [14,32,55]. Overall, the reports document an episodic and recurrent chronic course of illness, with recovery from index episode but frequent recurrence, and the presence of mood symptoms for most of time [14,27]. There is also evidence of heterogeneity in the severity of the course of illness at the individual patient level. In COBY, about one-fourth of the patients remained predominantly euthymic (i.e., most of the time had a euthymic mood) and another one-third were euthymic about half of the time [38]. On this point, however, not all the studies were concordant, with one study reporting that a large majority of the patients remained with clinically significant mood symptoms at the end of a four-year follow-up [16]. These differences in findings likely reflect heterogeneity in sample recruitment across the studies.

The high comorbidity with ADHD, anxiety, and substance abuse was also consistently documented [27,31,37,39], as well as the significant impairment in psychosocial and school functioning, despite stable cognitive functioning [42]. The persistence of mood symptoms, even at sub-syndromic levels, was associated with functional impairment [16].

The data also documented the high rate of suicidal behavior in youth with BD. A suicide attempt incidence of 14.7% over 3.5 years among child and adolescent BD patients [64] was reported, and a rate of 18–20% over a five-year period of observation [24,33]. This is consistent overall with the increased suicidal risk in adults with BD [1]. Consistent with the epidemiology of suicide attempt in the general population [65], in early onset BD the suicide attempt rate was also higher among female patients and associated with the presence and severity of depression [33].

Finally, the relevance of the psychosocial context to the course of BD emerged from several studies. Thus, improvement was faster in children living in low-conflict families, low maternal warmth was associated with higher risk of relapse [27,56], and suicidal risk was greater if the quality of family relationships was poor [51].

While the level of information provided by these reports is considerable, there are a number of limitations that leave important issues unaddressed. First, even if the continuity of mania/hypomania in adolescence into early adulthood is well-documented, no study extended beyond early adulthood. In particular, the course of early onset BD in adulthood remains unexplored with respect to social and occupational functioning. Second, although there are some indications that lithium may have beneficial effect by shortening time to recovery, reducing suicidality, and improving functioning [28,48], and that antimanic medication may decrease risk of recurrence [14], little information is available on the possible impact of early treatment on the long-term psychopathological trajectory of the disorder. The report that 28% of the patients relapsed despite being on apparently adequate doses of lithium is difficult to interpret without a comparison group [29]. Finally, given that most of the studies were conducted in the USA, there is a need for more research on clinical samples from other socio-cultural contexts.

Our review has several limitations. Only publications since 1990 were considered; however, the diagnosis of BD was infrequently applied to children and adolescents until the 1990s. The initial screening of the titles and abstracts of the publications identified by the electronic searches was conducted by multiple reviewers only for a sample of 600 publications (7% of the total), while the remaining publications were screened by just one reviewer. This might have resulted in missing relevant publications. However, the four reviewers achieved good agreement during their group reviews.

## 5. Conclusions

In conclusion, across a variety of studies, diverse for sample selection and assessment methods, there was evidence of substantial homotypic diagnostic continuity of early onset BD from adolescence into early adulthood, but with heterogeneity in the severity of the clinical course and still limited information about the distal impact of the disorder and its treatment on functional outcomes in adulthood.

## Figures and Tables

**Table 1:** Patient cohorts used to examine the course of bipolar disorder in children and adolescents.

	Study Cohorts	Design	Diagnostic Criteria	Size (n)	Mean Age at Intake, min-max (y)	Follow-Up (y)	Main Outcomes
1	Consecutive inpatients with BD-I at the University of California Los Angeles (UCLA) adolescent psychiatric unit Strober et al., 1995 [24]	Prospective research follow-up	RDC	54	16.0	5	96% recovered; 44% relapsed. Shorter recovery time if index episode was manic (median 8 weeks) or mixed (11 weeks) compared to depressed (26 weeks). 20% made a suicide attempt. Most common treatments: lithium, carbamazepine, antidepressants.
2	Patients with BD at the National Institute of Mental Health and Neurosciences, Bangalore, India, 1990–1991 Srinath et al., 1998 [25]	Prospective research follow-up	DSM-III-R	30	13.9	Up to 5	100% recovered; 67% relapsed.
3	First BD manic or mixed episode: the *Phenomenology and Course of Pediatric Bipolar Disorders* study at Washington University, St. Louis, MO, USA Geller et al., 2002 [26] Geller et al., 2004 [13] Geller, 2008 [27] Geller 2010 [28]	Prospective research follow-up	DSM-IV	115	11.1	Up to 8	88% recovered; 73% relapsed to mania [27]. Episode duration: mean 79.2 ± SD 66.7 weeks [13]. Time to recovery: mean 36.0 ± SD weeks at 2 y [26]. Time to relapse: mean 28.6 ± SD weeks at 2 y [26]. 60% of the time was spent with BD episodes (40% with mania) [27]. 63% were treated with antimanic medications [28]. More time on lithium predicted earlier recovery [28]. Living with intact biological family predicted recovery [26]. Low maternal warmth predicted relapse and more time with mania [27]. Younger age and psychosis predicted more time spent with mania [27]. 35% of patients above 18 y had substance use disorder [27].
4	Consecutive patients with BD-I at the National Institute of Mental Health and Neurosciences, Bangalore, India, 1997–2001 Rajeev et al., 2004 [29]	Medical record review	DSM-IV	139	13.1	4.7	100% recovered; 35% relapsed (13% despite therapeutic lithium levels). 89% of relapses were in the first 2 y.
5	Patients with BD diagnosis and multiple psychiatric hospitalizations at Harris County Psychiatric Center Pettit et al., 2005 [30]	Medical record review	DSM-III-R DSM-IV	70	13.5	9	73% diagnostic concordance between first and second hospitalization. BD had higher positive concordance than major depressive disorder.
6	Patients with BD-I, BD-II, or BD-NOS enrolled in the *Course and Outcome of Bipolar Youth* (COBY), at three university centers (Brown University, UCLA, and University of Pittsburgh, USA) Birmaher et al., 2006 [31] Birmaher et al., 2009 [14], Axelson et al., 2011 [32], Goldstein et al., 2012 [33], Goldstein et al., 2013 [34], Hower et al., 2013 [35], Hunt et al., 2013 [36], Sala et al., 2012 [37], Birmaher et al., 2014 [38], Sala et al., 2014 [39], Yen et al., 2015 [40], Borue et al., 2016 [41], Frías et al., 2017 [42], Krantz et al., 2018 [43], Hafeman et al., 2020 [17], Hower et al., 2019 [44], Pascual et al., 2019 [45], Shalev et al., 2019 [46], Frazier et al., 2020 [47], Hafeman et al. 2020 [48], Lee et al., 2020 [49], Mitchell et al., 2020 [50], Sewall et al., 2020 [51]	Prospective research follow-up	DSM-IV	446 enrolled (analyses on sample ranging from 135–446)	11.9 7–17	Up to 12.5 (analyses ranging from 2–12.5)	81.5% recovered within 2.5 y from index episode; 62.5% showed recurrence within 1.5 y [14]. Polarity of index episode predicted that of subsequent episodes [14]. At 4 y, 25% of BD-II had converted to BD-I, and 38% of BD-NOS to BD-I or BD-II. Predictors of conversion: family history of BD and prior psychiatric hospitalization. Predictors of non-conversion: antimanic medication treatment and history of psychotic symptoms [14,32]. Mood symptoms were present 60% of the time, with depressive being more common than manic symptoms [14]. Latent class growth analysis showed four mood trajectories: “predominantly euthymic” (24.0%), “moderately euthymic” (34.6%), “ill with improving course” (19.1%), and “predominantly ill” (22.3%). Within each class, youths were euthymic on average 84.4%, 47.3%, 42.8%, and 11.5% of the follow-up time, respectively [38]. Most patients (64%) had child-onset mania/hypomania and continued to experience these mood symptoms across adolescence and early adulthood [17]. Earlier onset, longer duration of mood symptoms, lower socioeconomic status, and psychosis were associated with poorer outcomes and rapid mood changes [31,38]. Males had earlier BD onset than females, but with no differences in rate or time to recovery or recurrence [50]. 18% attempted suicide over 5 y. Female sex, depression severity, and history of depression were predictors of suicide attempt [33]. Most patients presented both irritability and elation during follow-up [36]. Most patients had anxiety disorder that persisted over time and was associated with more mood recurrences and less time in euthymia [37,39]. 32% developed substance abuse disorder [34]. 12.2% had borderline personality disorder, which was associated with more severe affective instability [40]. Patients with both BD and autism spectrum disorder have earlier onset with mixed mood symptoms [41]. In transitioning to early adulthood (17–19 y), frequency of clinical visits decreased [35]. Hypomanic symptoms were associated with risky sexual behaviour [43]. More stable mood was associated with better psychosocial functioning, but some impairment was detected in 44% of the predominantly euthymic patients [44]. Patients with lifetime traumatic events had earlier BD onset, more severe symptoms, suicidal ideation, and worse psychosocial functioning [45]. Patients with lifetime psychosis had more severe mood and anxiety symptoms, higher suicidality, higher rates of psychiatric hospitalization and sexual/physical abuse, and worse psychosocial functioning [46]. After initial recovery, 40.5% still had functional impairment in interpersonal relationships and 92.8% in schoolwork. Decline in psychosocial functioning preceded depressive episodes [49]. Poor quality of the relationship with parents was a risk factor for suicide ideation, and recent worsening of the relationship increased risk of suicide attempt [51]. Cognitive functioning remained stable and in the normal range over a 2.5 y follow-up. Lower cognitive functioning was associated with more pervasive mood symptoms ad poorer functioning [42]. Increased energy level at intake did not influence distal clinical and functional outcomes [47]. Lithium therapy was associated with lower suicidality rates and better functioning [48].
7	Consecutive inpatients with BD-I manic/mixed episodes at the Psychiatric Units of Cincinnati Children’s Hospital Medical Center DelBello et al., 2007 [15]	Prospective research follow-up	DSM-IV	71	15.2	1.0	85% recovered. 39% reached functional recovery. 52% had recurrence (45% into mania, 39% into depression, and 16% into mixed state). Mean time to recovery was 20 weeks (SD 13). Comorbid ADHD was associated with longer time to recovery.
8	All patients with BD diagnosis in the Integrated Health Care Information Services (IHCIS) Managed Care Benchmark Database (USA) Castilla-Puentes, 2008 [52]	Managed care database	ICD-9	8129	12–18	1.4	58 (0.7%) had four or more episodes requiring hospitalization.
9	All patients with BD-I diagnosis admitted to public mental health centers in South Carolina, USA. Jerrell & Prewette, 2008 [53]	Medical record review	DSM-IV	82	6–17	1.5	40% with psychosis. 57% with suicidal ideation and/or behaviour. 69% with aggression. 28% with substance abuse. 56% were hospitalized at least once. 75% were treated pharmacologically: 56% with valproate and 43% with antipsychotic. 87% recovered. 64% relapsed.
10	All patients with BD diagnosis at child and adolescent psychiatry services of the University of Pamplona, Spain Escamilla et al., 2011 [54], Ribeiro-Fernandez et al., 2019 [55]	Medical record review	DSM-IV	72	12.6	Up to 15 Median: 3.9	95.8% retained BD diagnosis. At intake, 37.5% had BD-I, 8.3% BD-II, and 54.2% BD-NOS At follow-up, 62.5% had BD-I, 8.3% had BD-II, and 23.6% had BD-NOS All BD-I patients maintained their diagnosis. Half of all patients with baseline BD-NOS maintained their BD subtype, but most of the other half showed conversion to BD-I.
11	Patients with BD-I recruited at the Pediatric Psychopharmacology unit, Massachusetts General Hospital, Boston, MA, USA Wozniak et al., 2011 [16]	Prospective research follow-up	DSM-IV	78	13.4/6–17	4	73.1% continued to meet full diagnostic criteria for BD-I. 6.4% were euthymic at 4 y follow-up.
12	Patients with BD enrolled at Boulder, CO, and Pittsburgh, PA, USA Sullivan et al., 2012 [56]	Follow-up of participants in clinical trial	DSM-IV	58	14.5	2	Mania symptoms improved more rapidly in low-conflict than in high-conflict families. Family cohesion, adaptability, and conflict correlated with depression scores over time.
13	Patients with BD-I (n = 71) or other bipolar spectrum disorders (n = 91 with BD-II, BD-NOS, or cyclothymia) within the Longitudinal Assessment of Manic Symptoms (LAMS) study at four university clinics (Case Western Reserve University, Cincinnati Children’s Hospital Medical Center, Ohio State University, and University of Pittsburgh, USA) Findling et al., 2013 [57]	Prospective research follow-up	DSM-IV	162	6–12	2	73% of BD-I at intake showed reduction of manic symptoms over time. 24% of the patients with other bipolar spectrum disorders converted to BD-I.
14	All patients first diagnosed with BD under age 18 y in Denmark Kessing et al., 2015 [58] Laursen et al., 2020 [59]	Nationwide medical registry	ICD-10	Up to 519	15.9 y	Up to 10 y	Stability of BD diagnosis: 93% at 6 months, 86% at 3 y, and 73% at 10 y; 17% changed diagnosis to schizophrenia [59] Diagnostic change was more likely if the patient was male, diagnosed during hospitalization, demonstrated substance abuse, had a previous diagnosis of schizophrenia spectrum disorder, or had a family history of schizophrenia.
15	Patients with BD-I or BD-II enrolled at University of Colorado, Boulder, University of Pittsburgh, and Cincinnati Children’s Hospital Medical Center, USA Weintraub et al., 2020 [60]	Follow-up of participants in clinical trial	DSM-IV-TR	144	15.6 12–18	2	Latent class growth analyses indicated four mood trajectories: “predominantly euthymic” (29.9% of sample), “ill with significantly improving course” (11.1%), “moderately euthymic” (26.4%), and “ill with moderately improving course” (32.6%). More severe baseline depressive symptoms, suicidality, lower quality of life, and minority race/ethnicity predicted a clinical course with more severe mood symptoms.

BD-I: bipolar disorder type I; BD-II: bipolar disorder type II; BD-NOS: bipolar disorder not otherwise specified; COBY: Course and Outcome of Bipolar Youth; DSM: Diagnostic and Statistical Manual of Mental Disorders; editions: III, IV, IV-TR, and 5 (American Psychiatric Association 2013) [5,61]. ICD-10: International Classification of Diseases—10th edition [62]; RDC: Research Diagnostic Criteria (Spitzer et al., 1978) [63]; SD: standard deviation; y: year.

## Supplementary Materials

The following are available online at https://www.mdpi.com/2076-3425/11/3/341/s1. Table S1: Complete search string. Table S2: Risk of bias evaluation. Table S3: Publications used to examine the course of bipolar disorder in children and adolescents.
