# Supplementary material for: What Do We Know about the Long-Term Course of Early Onset Bipolar Disorder? A Review of the Current Evidence
Source: Brain Sci. 2021 Mar 8;11(3):341. doi: 10.3390/brainsci11030341 (PMC8001096; doi:10.3390/brainsci11030341)
Supplement: Supplementary file 1 [file brainsci-11-00341-s001.pdf]

Supplementary Table S1 - Complete search string.

**PubMed**	("Bipolar and Related Disorders"[Mesh] OR bipolar*[tiab] OR mania*[tiab] OR manic[tiab] OR hypomani*[tiab] OR hypo-mani*[tiab]) AND (juvenile*[tiab] OR COBY*[tiab] OR ((diagnosis[subheading] OR "Early Diagnosis"[Mesh] OR "Early Medical Intervention"[Mesh] OR "Age of Onset"[Mesh] OR diagnos*[tiab] OR onset*[tiab] OR phenomenol*[tiab]) AND ("Child"[Mesh] OR "Adolescent"[Mesh] OR child*[tiab] OR kid[tiab] OR kids[tiab] OR adolescen*[tiab] OR teen*[tiab] OR pediatr*[tiab] OR paediatr*[tiab] OR prepuber*[tiab] OR pre-puber*[tiab] OR youth[tiab] OR youths[tiab]))) AND ("Prognosis"[Mesh] OR "Disease Progression"[Mesh] OR prognos*[tiab] OR course[tiab] OR progression[tiab] OR "Epidemiologic Studies"[Mesh] OR "Observational Study"[Publication Type] OR observational-stud*[tiab] OR case-control*[tiab] OR case-comparison*[tiab] OR cohort*[tiab] OR longitudinal*[tiab] OR prospective*[tiab] OR retrospective*[tiab] OR cross-sectional*[tiab] OR transversal-stud*[tiab] OR follow-up[tiab] OR followup[tiab])
**Embase**	('mania'/exp OR bipolar*:ti,ab,kw OR mania*:ti,ab,kw OR manic:ti,ab,kw OR hypomani*:ti,ab,kw OR hypo-mani*:ti,ab,kw) AND ('juvenile'/de OR juvenile*:ti,ab,kw OR COBY*:ti,ab,kw OR ((diagnosis:lnk OR 'diagnosis'/exp OR 'early intervention'/exp OR 'onset age'/exp OR diagnos*:ti,ab,kw OR onset*:ti,ab,kw OR phenomenol*:ti,ab,kw) AND ('child'/exp OR 'adolescent'/exp OR child*:ti,ab,kw OR kid:ti,ab,kw OR kids:ti,ab,kw OR adolescen*:ti,ab,kw OR teen*:ti,ab,kw OR pediatr*:ti,ab,kw OR paediatr*:ti,ab,kw OR prepuber*:ti,ab,kw OR pre-puber*:ti,ab,kw OR youth:ti,ab,kw OR youths:ti,ab,kw))) AND ('disease course'/exp OR 'treatment outcome'/exp OR prognos*:ti,ab,kw OR course:ti,ab,kw OR progression:ti,ab,kw OR 'observational study'/exp OR 'case control study'/exp OR 'cohort analysis'/exp OR 'longitudinal study'/exp OR 'prospective study'/exp OR 'retrospective study'/exp OR 'cross-sectional study'/exp OR 'follow up'/exp OR observational-stud*::ti,ab,kw OR case-control*:ti,ab,kw OR case-comparison*:ti,ab,kw OR cohort*:ti,ab,kw OR longitudinal*:ti,ab,kw OR prospective*:ti,ab,kw OR retrospective*:ti,ab,kw OR cross-sectional*:ti,ab,kw OR transversal-stud*:ti,ab,kw OR follow-up:ti,ab,kw OR followup:ti,ab,kw)

**PsycINFO**	(DE "Bipolar Disorder" OR DE "Bipolar I Disorder" OR DE "Bipolar II Disorder" OR DE "Cyclothymic Disorder" OR DE "Mania" OR TI bipolar* OR AB bipolar* OR TI mania* OR AB mania* OR TI manic OR AB manic OR TI hypomani* OR AB hypomani* OR TI hypo-mani* OR AB hypo-mani*) AND (TI juvenile* OR AB juvenile* OR TI COBY* OR AB COBY* OR ((DE "Diagnosis" OR DE "Computer Assisted Diagnosis" OR DE "Differential Diagnosis" OR DE "Dual Diagnosis" OR DE "Medical Diagnosis" OR DE "Psychodiagnosis" OR DE "Early Intervention" OR DE "Onset (Disorders)" OR TI diagnos* OR AB diagnos* OR TI onset* OR AB onset* OR TI phenomenol* OR AB phenomenol*) AND (TI child* OR AB child* OR TI kid OR AB kid OR TI kids OR AB kids OR TI adolescen* OR AB adolescen* OR TI teen* OR AB teen* OR TI pediatr* OR AB pediatr* OR TI paediatr* OR AB paediatr* OR TI prepuber* OR AB prepuber* OR TI pre-puber* OR AB pre-puber* OR TI youth OR AB youth OR TI youths OR AB youths))) AND (DE "Prognosis" OR DE "Disease Course" OR DE "Disease Progression" OR TI prognos* OR AB prognos* OR TI course OR AB course OR TI progression OR AB progression OR TI "observational study" OR AB "observational study" OR TI "observational studies" OR AB "observational studies" OR TI "case-control" OR AB "case-control" OR TI "case-controlled" OR AB "case-controlled" OR TI "case-comparison" OR AB "case-comparison" OR TI "case-comparisons" OR AB "case-comparisons" OR TI cohort* OR AB cohort* OR TI longitudinal* OR AB longitudinal* OR TI prospective* OR AB prospective* OR TI retrospective* OR AB retrospective* OR TI "cross-sectional" OR AB "cross-sectional" OR TI "transversal study" OR AB "transversal study" OR TI "transversal studies" OR AB "transversal studies" OR TI "follow-up" OR AB "follow-up" OR TI followup OR AB followup)

Supplementary Table S2 - Risk of Bias Evaluation

Study:

Criteria	Risk of bias		
	Low	Moderate	High
Representativeness of study sample (external validity)			
Validated diagnostic interviews			
Blind raters (internal validity)			
Adequate sample size (internal validity)			
Sample retention (low: >80%; moderate: 60-80%; high: <60%)			

Supplementary Table S3 - Publications used to examine the course of bipolar disorder in children and adolescents

	Publications	Number of patients	Mean age at diagnosis (y)	Duration of Follow up (y)	Main topic	*** Risk-of-Bias Evaluation H: High M: Moderate L: Low
1	Michael Strober et al., «Recovery and Relapse in Adolescents with Bipolar Affective Illness: A Five-Year Naturalistic, Prospective Follow-Up», *Journal of the American Academy of Child & Adolescent Psychiatry* 34, n. 6 (giugno 1995): 724–31 [[24]]	54	16.0	5	course of disease	H (C)
2	S. Srinath et al., «A Prospective Study of Bipolar Disorder in Children and Adolescents from India», *Acta Psychiatrica Scandinavica* 98, n. 6 (dicembre 1998): 437–42 [[25]]	30	13.9	4-5	course of disease, comorbidity	H (D)
3	Barbara Geller et al., «Two-Year Prospective Follow-Up of Children With a Prepubertal and Early Adolescent Bipolar Disorder Phenotype», *American Journal of*	89	10.9	2	course of disease	M (D)

	Psychiatry 159, n. 6 (giugno 2002): 927–33 [26]					
4	Barbara Geller et al., «Four-Year Prospective Outcome and Natural History of Mania in ChildrenWith a Prepubertal and Early Adolescent Bipolar Disorder Phenotype», *Archives of General Psychiatry* 61, n. 5 (1 maggio 2004): 459 [13]	86	10.8	4	course of disease	M (D)
5	J Rajeev et al., «A Systematic Chart Review of the Naturalistic Course and Treatment of Early-Onset Bipolar Disorder in a Child and Adolescent Psychiatry Center», *Comprehensive Psychiatry* 45, n. 2 (marzo 2004): 148–54 * [29]	139	13.1	4.6	course of disease,<br><br>treatment	H (C)
6	Jeremy W. Pettit, Sharon Morgan, e Amber L. Paukert, «The Stability of Axis I Diagnoses in Youth Across Multiple Psychiatric Hospitalizations», *Child Psychiatry and Human Development* 36, n. 1	815 of which 19+51 subjects with BD at intake.	13.5	9	diagnostic stability	H (B)

	(settembre 2005): 53–71* [30]					
7	Boris Birmaher et al., «Clinical Course of Children and Adolescents With Bipolar Spectrum Disorders», *ARCH GEN PSYCHIATRY* 63 (2006): 10 [31]	263	13	2	course of disease	H (C)
8	Melissa P DelBello et al., «Twelve-Month Outcome of Adolescents With Bipolar Disorder Following First Hospitalization for a Manic or Mixed Episode», *Am J Psychiatry*, 2007, 9 [15]	71	15.2	1	course of disease, comorbidity, treatment	H (C)
9	Ruby Castilla-Puentes, «Multiple Episodes in Children and Adolescents with Bipolar Disorder: Comorbidity, Hospitalization, and Treatment (Data from a Cohort of 8,129 Patients of a National Managed Care Database)», *The International Journal of Psychiatry in Medicine* 38, n. 1 (marzo 2008): 61–70* [52]	8129	80% between 12-18 y	3	course of disease, comorbidity, treatment	H (B) H (C)

10	Barbara Geller et al., «Child Bipolar I Disorder: Prospective Continuity With Adult Bipolar I Disorder; Characteristics of Second and Third Episodes; Predictors of 8-Year Outcome», *Archives of General Psychiatry* 65, n. 10 (6 ottobre 2008): 1125 [[27]]	115	11.1	8	comorbidity,<br><br>course of disease,<br><br>diagnostic stability	L
11	Jeanette M. Jerrell e Ervin D. Prewette, «Outcomes for Youths with Early- and Very-Early-Onset Bipolar I Disorder», *The Journal of Behavioral Health Services & Research* 35, n. 1 (gennaio 2008): 52–59* [[63]]	82	6-17	1.5	course of disease,<br><br>treatment,<br><br>suicide,<br><br>comorbidity	H (B)<br><br>H (C)
12	Boris Birmaher et al., «Four-Year Longitudinal Course of Children and Adolescents With Bipolar Spectrum Disorders: The Course and Outcome of Bipolar Youth (COBY) Study», *Am J Psychiatry*, 2009, 10 [[14]]	413	12.6	4	course of disease,<br><br>diagnostic stability	M (C)
13	Barbara Geller et al., «Pharmacological and Non-Drug Treatment of Child Bipolar I Disorder during Prospective Eight-Year Follow-Up», *Bipolar*	115	11.1	8	treatment	L

	Disorders 12, n. 2 (marzo 2010): 164–71 [28]					
14	David A. Axelson et al., «Course of Subthreshold Bipolar Disorder in Youth: Diagnostic Progression From Bipolar Disorder Not Otherwise Specified», *Journal of the American Academy of Child & Adolescent Psychiatry* 50, n. 10 (ottobre 2011): 1001-1016.e3 [32]	140	11.9	5	diagnostic stability,<br><br>treatment	M (C)
15	Inmaculada Escamilla et al., «Pediatric Bipolar Disorder in a Spanish Sample: Results after 2.6years of Follow-Up», *Journal of Affective Disorders* 132, n. 1–2 (luglio 2011): 270–74 * [53]	38	13.9	2.6	course of disease,<br><br>comorbidity	H (C)<br><br>H (D)
16	Janet Wozniak et al., «High Level of Persistence of Pediatric Bipolar-I Disorder from Childhood onto Adolescent Years: A Four Year Prospective Longitudinal Follow-up Study», *Journal of Psychiatric Research* 45,	78	13.4	4	diagnostic stability,cours e of<br><br>disease,<br><br>comorbidity	M (A)<br><br>M (D)<br><br>M (E)

	n. 10 (ottobre 2011): 1273–82 [16]					
17	Tina R. Goldstein et al., «Predictors of Prospectively Examined Suicide Attempts Among Youth With Bipolar Disorder», *Archives of General Psychiatry* 69, n. 11 (1 novembre 2012) [33]	413	12.6	5	suicide	M (C)
18	Regina Sala et al., «Factors Associated With the Persistence and Onset of New Anxiety Disorders in Youth With Bipolar Spectrum Disorders», *The Journal of Clinical Psychiatry* 73, n. 01 (15 gennaio 2012): 87–94 [37]	413	12.6	5	comorbidity	M (C)
19	Aimee E. Sullivan et al., «Family Functioning and the Course of Adolescent Bipolar Disorder», *Behavior Therapy* 43, n. 4 (dicembre 2012): 837–47** [55]	58	14.5	2	functioning	H (C)
20	Robert L Findling et al., «The 24-Month Course of Manic Symptoms in Children», *Bipolar Disorders* 15, n. 6	621	6-12	2	diagnostic stability	L

	(settembre 2013): 669–79 [56]					
21	Benjamin I. Goldstein et al., «Predictors of First-Onset Substance Use Disorders During the Prospective Course of Bipolar Spectrum Disorders in Adolescents», *Journal of the American Academy of Child & Adolescent Psychiatry* 52, n. 10 (ottobre 2013): 1026–37 [34]	167	15.5	4	comorbidity, treatment	M (C)
22	Heather Hower et al., «Use of Mental Health Services in Transition Age Youth with Bipolar Disorder»:, *Journal of Psychiatric Practice* 19, n. 6 (novembre 2013): 464–76 [35]	413	12.6	4	treatment	M (C)
23	Jeffrey I. Hunt et al., «Irritability and Elation in a Large Bipolar Youth Sample: Relative Symptom Severity and Clinical Outcomes Over 4 Years», *The Journal of Clinical Psychiatry* 74, n. 01 (15 gennaio 2013): e110–17 [36]	309	7-17	4	course of disease	M (C)

24	Boris Birmaher et al., «Longitudinal Trajectories and Associated Baseline Predictors in Youths With Bipolar Spectrum Disorders», *American Journal of Psychiatry* 171, n. 9 (settembre 2014): 990–99 [[38]]	367	12.6	4	diagnostic stability	M (C)
25	Regina Sala et al., «Effects of Comorbid Anxiety Disorders on the Longitudinal Course of Pediatric Bipolar Disorders», *Journal of the American Academy of Child & Adolescent Psychiatry* 53, n. 1 (gennaio 2014): 72–81 [[39]]	413	12.6	5	comorbidity	M (C)
26	Lars Vedel Kessing, Eleni Vradi, e Per Kragh Andersen, «Diagnostic Stability in Pediatric Bipolar Disorder», *Journal of Affective Disorders* 172 (febbraio 2015): 417–21* [[57]]	354	17.4	16	diagnostic stability,<br><br>comorbidity	H (B)<br><br>H (C)
27	S. Yen et al., «Borderline Personality Disorder in Transition Age Youth with Bipolar Disorder», *Acta Psychiatrica Scandinavica* 132, n. 4 (ottobre 2015): 270–80 [[40]]	271	13.0	7.7	comorbidity,<br><br>suicide	M (C)

28	Xenia Borue et al., «Longitudinal Course of Bipolar Disorder in Youth With High-Functioning Autism Spectrum Disorder», *Journal of the American Academy of Child & Adolescent Psychiatry* 55, n. 12 (dicembre 2016): 1064-1072.e6 [[41]]	368	7-17	9	comorbidity	M (C)
29	Megan Krantz et al., «Sexual Risk Behavior Among Youth With Bipolar Disorder: Identifying Demographic and Clinical Risk Factors», *Journal of the American Academy of Child & Adolescent Psychiatry* 57, n. 2 (febbraio 2018): 118–24 [[43]]	413	12.6	9.7	functioning	M (C)
30	Álvaro Frías et al., «Longitudinal Cognitive Trajectories and Associated Clinical Variables in Youth with Bipolar Disorder», *Bipolar Disorders* 19, n. 4 (giugno 2017): 273–84 [[42]]	135	16.1	2.5	course of disease,<br><br>functioning	M (C)

31	Heather Hower et al., «Predictors of Longitudinal Psychosocial Functioning in Bipolar Youth Transitioning to Adults», *Journal of Affective Disorders* 246 (marzo 2019): 578–85 [44]	367	7-17	11.5	functioning	M (C)
32	María Ribeiro-Fernández, Azucena Díez-Suárez, e César Soutullo, «Phenomenology and Diagnostic Stability of Paediatric Bipolar Disorder in a Spanish Sample», *Journal of Affective Disorders* 242 (gennaio 2019): 224–33* [54]	72	12.6	15	diagnostic stability	M (C)<br>M (D)
33	Amit Shalev et al., «Longitudinal Course and Risk Factors Associated with Psychosis in Bipolar Youths», *Bipolar Disorders* 22, n. 2 (marzo 2020): 139–54 [46]	370	12.6 (non psychosis) , 12.7 (psychosis)	11.7	comorbidity,<br>suicide,<br>functioning	M (C)
34	Elisabeth A. Frazier et al., «Correlates, Course, and Outcomes of Increased Energy in Youth with Bipolar Disorder», *Journal of Affective Disorders* 271 (giugno 2020): 248–54 [47]	398	13	12.5	diagnostic stability,<br>functioning	M (C)

35	Danella M. Hafeman et al., «Lithium Versus Other Mood-Stabilizing Medications in a Longitudinal Study of Youth Diagnosed With Bipolar Disorder», *Journal of the American Academy of Child & Adolescent Psychiatry* 59, n. 10 (ottobre 2020): 1146–55 [[48]]	340	12.6	10	treatment	M (C)
36	Danella M. Hafeman et al., «Prospectively Ascertained Mania and Hypomania Among Young Adults with Child- and Adolescent-Onset Bipolar Disorder», *Bipolar Disorders*, 19 dicembre 2020, bdi.13034 [[17]]	297	12.8	13.3	diagnostic stability	M (C)
37	Mathilde Frahm Laursen et al., «Diagnostic Stability in Children and Adolescents with Bipolar Disorder, a Nationwide Register-Based Study», *International Journal of Bipolar Disorders* 8, n. 1 (dicembre 2020): 14* [[58]]	519	15.9	10 from index	diagnostic stability,<br><br>comorbidity	H (B)<br><br>H (C)
38	Erica J. Lee et al., «Course of Longitudinal Psychosocial Functioning in Bipolar Youth Transitioning to Adults»,	367	12.6	4	functioning	M (C)

	Journal of Affective Disorders 268 (maggio 2020): 109–17 [49]					
39	Rachel H. B. Mitchell et al., «Sex Differences in the Longitudinal Course and Outcome of Bipolar Disorder in Youth», *The Journal of Clinical Psychiatry* 81, n. 6 (27 ottobre 2020) [50]	370	12.7	6	Course of disease	M (C)
40	Maria Andreu Pascual et al., «The Effect of Traumatic Events on the Longitudinal Course and Outcomes of Youth with Bipolar Disorder», *Journal of Affective Disorders* 274 (settembre 2020): 126–35 [45]	375	17	8.7	comorbidity,<br>suicide,<br>course of disease,<br>functioning	M (C)
41	Craig J.R. Sewall et al., «A Bayesian Multilevel Analysis of the Longitudinal Associations between Relationship Quality and Suicidal Ideation and Attempts among Youth with Bipolar Disorder», *Journal of Child Psychology and Psychiatry*, 27 ottobre 2020, jcpp.13343, [51]	386	11	12	suicide,<br>functioning	M (C)

42	Marc J. Weintraub et al., «Classifying Mood Symptom Trajectories in Adolescents With Bipolar Disorder», *Journal of the American Academy of Child & Adolescent Psychiatry* 59, n. 3 (marzo 2020): 381–90** [59]	144	15.6	2	course of disease,<br>suicide,<br>functioning	H (A)<br>H (C)

* medical records

**clinical trials

*** **Risk-of-Bias Evaluation**:

A. Representativeness of study sample (external validity)

B. Validated diagnostic interviews

C. Blind raters (internal validity)

D. Adequate sample size (internal validity) (low: n > 100; moderate: n= 50-100; high n < 50)

E. Sample retention (low: >80%; moderate: 60-80%; high: <60%)
